# Biological and pharmacological functions of the FGF19- and FGF21-coreceptor beta klotho

**DOI:** 10.3389/fendo.2023.1150222

**Published:** 2023-05-16

**Authors:** Alexandra S. Aaldijk, Cristy R. C. Verzijl, Johan W. Jonker, Dicky Struik

**Affiliations:** Section of Molecular Metabolism and Nutrition, Department of Pediatrics, University of Groningen, University Medical Center Groningen, Groningen, Netherlands

**Keywords:** beta klotho, FGF19, FGF21, FGFR, NAFLD, NASH, diabetes, metabolism

## Abstract

Beta klotho (KLB) is a fundamental component in fibroblast growth factor receptor (FGFR) signaling as it serves as an obligatory coreceptor for the endocrine hormones fibroblast growth factor 19 (FGF19) and fibroblast growth factor 21 (FGF21). Through the development of FGF19- and FGF21 mimetics, KLB has emerged as a promising drug target for treating various metabolic diseases, such as type 2 diabetes (T2D), non-alcoholic fatty liver disease (NAFLD), and cardiovascular disease. While rodent studies have significantly increased our understanding of KLB function, current clinical trials that test the safety and efficacy of KLB-targeting drugs raise many new scientific questions about human KLB biology. Although most KLB-targeting drugs can modulate disease activity in humans, individual patient responses differ substantially. In addition, species-specific differences in KLB tissue distribution may explain why the glucose-lowering effects that were observed in preclinical studies are not fully replicated in clinical trials. Besides, the long-term efficacy of KLB-targeting drugs might be limited by various pathophysiological conditions known to reduce the expression of KLB. Moreover, FGF19/FGF21 administration in humans is also associated with gastrointestinal side effects, which are currently unexplained. A better understanding of human KLB biology could help to improve the efficacy and safety of existing or novel KLB/FGFR-targeting drugs. In this review, we provide a comprehensive overview of the current understanding of KLB biology, including genetic variants and their phenotypic associations, transcriptional regulation, protein structure, tissue distribution, subcellular localization, and function. In addition, we will highlight recent developments regarding the safety and efficacy of KLB-targeting drugs in clinical trials. These insights may direct the development and testing of existing and future KLB-targeting drugs.

## 1 Introduction

The identification of the *KLB* gene was preceded by the discovery of a homologous gene designated klotho (α-klotho or *Kl*) that was found to be involved in aging (1). Deficiency of the *Kl* gene in mice results in a syndrome that resembles human aging, characterized by a short lifespan, infertility, atherosclerosis, skin atrophy, osteoporosis, and emphysema (2). Around a decade later, it was demonstrated that KL serves as an obligatory coreceptor for FGF23, a member of the FGF superfamily (3). The connection between KL and FGF23 was partly made by the observation that *Fgf23*- and *Kl*-deficient mice have a similar complex aging phenotype (1). Currently, the FGF23-KL axis is mainly known for regulating phosphate homeostasis, which is achieved through increasing urinary phosphate excretion and lowering levels of the mineral-regulating hormones parathyroid hormone and vitamin D (1). The role of the FGF23-KL axis in controlling phosphate homeostasis also partly explains the premature aging of *Fgfr23-* and *Kl*-deficient mice, as aging symptoms are reduced by consuming a low-phosphate diet (4).

A homology search using the *Kl* sequence led to the identification of a partial cDNA clone in the GenBank database (5). Using primers designed from this partial cDNA clone, the full-length mouse cDNA clone was isolated, which showed 41.2% amino acid sequence similarity to KL and was therefore termed beta klotho *(KLB)* (5). The subsequent generation of a *Klb*-deficient mouse model showed that these mice are characterized by a strongly elevated synthesis and excretion of bile acids (6). In these mice, suppression of cholesterol 7α-hydroxylase (*Cyp7a1*), the rate-limiting enzyme in bile acid (BA) synthesis, was substantially impaired (6). Strong phenotypic similarities between *Fgfr4-, Klb-*, and *Fgf15-*deficient mice such as high BA production and increased *Cyp7a1* expression, led to the idea that this complex acts as a negative feedback loop in BA synthesis through transcriptional repression of *Cyp7a1* (6–8). Not much later, the concept that KLB may serve as a coreceptor in FG15/FGFR4 signaling, similar to the role of KL in FGF23/FGFR signaling, was put forward and confirmed (9, 10). In addition, around the same period, it was shown that the activity of a related FGF member, FGF21, also depended on KLB (9). Based on these scientific milestones, the *KLB* gene has now been identified to encode a single-pass transmembrane protein that functions as a high-affinity receptor for FGF19 and FGF21.

The co-factor requirement of FGF19, FGF21, and FGF23 set these FGFs apart from canonical FGF family members. The FGF family comprises 22 closely related proteins that are secreted from cells (11). Functional classification of FGF proteins shows that most members of the FGF family function as autocrine/paracrine hormones. Upon secretion, autocrine/paracrine FGFs bind to heparan sulfate proteoglycans (HSPG), which limits their diffusion into the interstitial space and regulates their activity. In addition, the three endocrine FGFs, FGF19, FGF21, and FGF23, have lost their ability to bind HSPG and can diffuse into the bloodstream. Instead of binding to HSPG, endocrine FGFs evolved to bind KL (FGF23) or KLB (FGF19 and FGF21). The binding of FGFs to HSPG or KL/KLB facilitates their interaction with FGF receptors (FGFRs) (12). Four *FGFR* genes (*FGFR1-4*) have been identified in humans and mice. The binding of FGF ligands to FGFRs results in receptor stimulation and downstream activation of at least four canonical signaling pathways, including MAPK, PI3K, PLCγ, and STAT. Ultimately, altered FGFR activity is linked to the modulation of a wide range of cellular responses, such as growth, proliferation, differentiation, migration, and apoptosis (13).

While most FGFs play a role in embryonic development, FGF15/FGF19 and FGF21 emerged as critical regulators of metabolic homeostasis during adulthood (8, 14). Subsequent preclinical studies have shown that pharmacological administration of recombinant FGF19 or FGF21, or antibodies directed against KLB/FGFR have potent, partially overlapping metabolic effects, including the lowering of blood glucose levels, hepatic fat content, and plasma bile acid and cholesterol levels, highlighting their potential as new drug candidates for the treatment of various obesity-related disorders, including T2D, NAFLD, cardiovascular disease, and cholestasis (15). The metabolic effects of FGFs appear to be primarily driven by selective targeting of KLB/FGFR4 in the liver and KLB/FGFR1 in the adipose tissue and certain brain areas (16). Although rodent studies have contributed significantly to our understanding of FGF19/FGF21 and KLB function, clinical trials testing the safety and efficacy of KLB-targeting drugs raise the question of how these biologicals act in humans. Given the central role of KLB in determining the specificity and activity of FGF-mimetics, a better understanding of human KLB function is urgently needed. In this review, we describe the current knowledge of KLB biology, including its genetic variants, transcriptional regulation, protein structure, tissue distribution, subcellular localization, and function.

## 2 KLB: from gene to protein

### 2.1 Genetic *KLB* variants and their phenotypic associations

The *KLB* gene is localized on chromosome 4 of the human genome and is composed of 5 exons, spanning a total length of 44.604 base pairs. Within this genetic locus, whole-exome sequencing and whole-genome sequencing studies have identified thousands of genetic variants, of which the functional and pathophysiological consequences remain largely unknown. The Genome Aggregation Database (gnomAD), comprised of aggregated data from 125,748 exomes and 15,708 genomes, reports 980 germline *KLB* gene variants, counting 251 synonymous, 477 missense, and 12 loss-of-function (LoF) variants (17). Interestingly, the number of observed LoF variants is around four times lower than the expected number of LoF variants (12 *vs*. 45.7) within this gene, with no homozygous LoF carriers identified in this cohort. Similarly, the observed number of missense variants is also lower than the number of expected missense variants (477 *vs*. 607.6), with only 16 of the 477 missense variants present in a homozygous state. In contrast, the expected and observed numbers of synonymous variants in the *KLB* gene are similar (258.2 *vs*. 251). These findings suggest that the *KLB* gene is under selection against LoF and missense variants, indicating that harmful *KLB* variants are removed from the population by natural selection.

The Trans-Omics for Precision Medicine (TOPMed) database, which provides genetic information observed in 132,345 sequenced genomes, reports similar numbers of synonymous (220), missense (426), and LoF (33) variants in the *KLB* gene compared to the gnomAD (18). However, due to a 10-fold greater number of completely sequenced genomes in TOPMed compared to gnomAD, the total number of intergenic variants in the TOPMed database is much higher, bringing the total number of *KLB* variants to more than 10,000. Similar to gnomAD, no homozygous LoF carriers were observed in TOPMed. Also, only 12 out of 426 missense variants are in a homozygous state. Thus, both gnomAD and TOPMed show that the *KLB* gene is genetically constrained, and that complete loss of both alleles is extremely rare. Consequently, sequencing larger numbers of genomes, as currently performed by the UK Biobank, which is expected to release genetic data of all 500,000 participants at the end of 2023, may reveal if homozygous LoF carriers exist.

#### 2.1.1 Phenotypic associations of the KLB variant Arg728Gln

Although the functional and clinical relevance of genetic variation in the *KLB* gene remains largely unclear, several studies have reported associations between *KLB* variants and phenotypic outcomes or disease (**Table 1**). To understand the genetic factors contributing to BA malabsorption in diarrhea-predominant irritable bowel syndrome (IBS-D), Wong et al. tested the association of 15 common single nucleotide polymorphisms (SNPs) from 7 genes critical to BA metabolism with symptom-based subgroups (19). SNP rs17618244 (Arg728Gln in KLB) was associated with colonic transit at 24 hours. Specifically, the G allele (Arg728) was associated with increased colonic transit compared to the A allele (Gln728). In addition, interaction tests revealed that two missense variants in *FGFR4* (Val10Ile and Gly388Arg) significantly affected the association of Arg728Gln with colonic transit. Furthermore, functional analysis of these *KLB* variants showed that Arg728 reduced KLB protein stability compared to Gln728 (19). Reduced protein stability of the Arg728 variant might weaken FGF19 binding and signaling, resulting in increased CYP7A1 expression and elevated BA synthesis that eventually causes an accelerated colonic transit (19).

**Table 1:** Overview of *KLB* gene variants and their phenotypic associations reported in the literature.

KLB variant	Functional assay	Cell line	Phenotype association	Study
Arg728Gln	↑ Protein stability	HEK293	Colonic transit at 24 hours	Wong et al., 2011 (19).
Arg728Gln	↓ Protein expression ↑ Lipid accumulation	HepG2 Huh7	Ballooning and lobular inflammation in pediatric NAFLD	Dongiovanni et al., 2020 (20).
Arg728Gln	↓ Protein expression ↑ Pro-fibrogenic genes induction ↑ Cell proliferation	LX-2	Lobular inflammation and hepatic fibrosis in MAFLD	Panera et al., 2021 (21).
Arg309Trp	↓ FGF21 signaling, ↓ Cell surface expression, ↓ Expression, ↓ Maturation level	L6 myoblasts	CHH	Xu et al., 2017 (22).
Arg309Gln	↓ FGF21 signaling ↓ Cell surface expression	L6 myoblasts	CHH	Xu et al., 2017 (22).
Arg424Cys	↓ FGF21 signaling ↓ Maturation level	L6 myoblasts	CHH	Xu et al., 2017 (22).
Ala574Thr	↓ Cell surface expression ↓ Maturation level	L6 myoblasts	CHH	Xu et al., 2017 (22).
Phe777delPhe	↓ FGF21 signaling ↓ Cell surface expression	L6 myoblasts	CHH	Xu et al., 2017 (22).
Lys815Glu	↓ FGF21 signaling ↓ Cell surface expression ↓ Maturation level	L6 myoblasts	CHH	Xu et al., 2017 (22).
Leu1011Pro	↓ FGF21 signaling ↓ Cell surface expression ↓ Maturation level	L6 myoblasts	CHH	Xu et al., 2017 (22).
Ser9Tyr (digenic with Val102Ile in *FGFR1*)	↓ FGF21 stimulated ERK phosphorylation	L6 myoblasts	Insulin-mediated pseudoacromegaly	Stone et al., 2020 (23).

CHH, congenital hypogonadotrophic hypogonadism; NAFLD, non-alcoholic fatty liver disease; MAFLD, metabolic-associated fatty liver disease; FGF21, fibroblast growth factor 21; ERK, extracellular signal-regulated kinases.

More recently, two other studies found that the Arg728Gln *KLB* variant is also associated with liver damage in children and adults with NAFLD (20, 21). Because the FGF19 pathway and bile acids are suggested to play a role in the pathogenesis of NAFLD (24, 25), Dongiovanni et al. addressed the impact of the Arg728Gln *KLB* variant on liver damage in 249 pediatric patients with biopsy-proven NAFLD (20). In this study, Arg728Gln was linked to an increased risk of ballooning and lobular inflammation. Overexpression of Arg728Gln in HepG2 and Huh7 cells decreased KLB protein expression, increased intracellular lipid accumulation, and caused an upregulation of lipotoxic and inflammatory genes (20). Another study from the same group examined the impact of Arg728Gln on liver damage in 1111 adults with NAFLD, showing that Arg728Gln was associated with fibrosis and lobular inflammation but not with steatosis (21). Similar to their previous study, overexpression of Arg728Gln decreased KLB protein levels in LX-2 cells. In addition, Arg728Gln overexpression induced pro-fibrogenic genes and enhanced LX-2 proliferation (21). Decreased KLB protein expression due to the substitution of Arg to Gln in these studies conflicts with the study of Wong et al., who showed that Arg728, and not Gln728, decreased protein stability (19). Therefore, additional functional analyses are needed to demonstrate how this variant behaves in different cellular contexts and to what extent it affects downstream FGFR signaling and cellular function.

#### 2.1.2 Phenotypic associations of other KLB variants

While the association of *KLB* variants with colonic transit and liver damage fits well with the known function of KLB as a critical regulator of bile acid homeostasis, other *KLB* variants were linked with phenotypes less clearly connected to bile acid metabolism, including alcohol intake, congenital hypogonadotrophic hypogonadism (CHH), and insulin-mediated pseudoacromegaly (IMPA) (22, 23, 26). In a genome-wide association meta-analysis and replication study among >105,000 individuals, a variant located in intron 1 of the *KLB* gene (rs11940694, A>G) was associated with alcohol consumption (26). A link between alcohol intake and KLB is supported by the observation that ethanol is one of the most potent inducers of plasma FGF21 in humans (27–29). In rodents and non-human primates, various mechanistic studies have also demonstrated causal effects between FGF21/KLB and alcohol intake. Administration of recombinant FGF21 or its transgenic overexpression reduced alcohol preference in mice, an effect that was absent in mice with a brain-specific deficiency of KLB (26). Similarly, the administration of the FGF21 analog PF-05231023 reduced alcohol consumption in non-human primates (30, 31). Mechanistically, FGF21 targets KLB-expressing neurons in the basolateral amygdala, which project to the nucleus accumbens to lower alcohol intake (30). Besides regulation of alcohol intake, FGF21 has also been reported to counteract alcohol intoxication by activating noradrenergic neurons in the locus coeruleus, a brain region controlling arousal and alertness (32). As plasma ethanol clearance was not affected in liver-specific FGF21- or neuron-specific KLB-deficient mice, the FGF21-KLB axis is suggested to counteract alcohol intoxication without modulating alcohol catabolism (32). Interestingly, Cas9-mediated knockout (KO) of *KLB* in human hepatocyte cell lines does modulate the expression of genes involved in alcohol metabolism, revealing potential species differences in KLB-dependent regulation of peripheral alcohol metabolism (33).

CHH is a rare disorder characterized by the absence or delay of puberty and infertility due to gonadotropin-releasing hormone (GnRH) deficiency. CHH can be caused by mutations in more than 30 genes (22). *FGFR1* is the most frequently mutated gene in CHH and participates in developing and maintaining GnRH neurons. Previous studies have shown that the *FGFR1* mutation Leu342Ser impaired FGFR1-KLB interaction and reduced FGF21 signaling. Therefore, mutations in *KLB* might be involved in CHH. Xu et al. performed a genetic screening on 334 CHH patients and identified seven heterozygous *KLB* variants in 13 patients (22). The functional effects of these *KLB* variants were determined in various cell-based assays (**Table 1**). All *KLB* variants, except Arg309Trp, demonstrated reduced maximal FGF21 response compared to wild-type KLB. In contrast, Arg309Trp showed a decreased EC_50,_ indicating reduced FGF21 affinity. Co-immunoprecipitation studies revealed that all *KLB* variants exhibited regular binding to FGFR1c. Similarly, the KLB variants did not affect KLB protein levels. In contrast, the Arg309Trp, Arg424Cys, K815E, and L1011P variants were characterized by altered glycosylation. In addition, Arg309Trp, Arg309Gln, Phe777delPhe, Lys815Glu, and Leu1011Pro variants exhibited reduced cell surface expression compared to wildtype KLB. Therefore, impaired functionality was demonstrated for all *KLB* variants. Furthermore, according to guidelines of the American College of Medical Genetics and Genomics (ACMG), all *KLB* variants were classified as pathogenic or likely pathogenic, except for Leu1011Pro, which was classified as a variant of uncertain significance (VUS). Support for the role of KLB in regulating puberty and fertility was provided by showing that deficiency of *Klb* in mice led to delayed puberty, altered estrous cyclicity, and subfertility. In addition, FGF21-stimulated release of GnRH from GnRH neurons derived from *Klb*-KO mice was strongly blunted (22).

Finally, digenic variants in *FGFR1* and *KLB* have been reported in a case study involving a 12-year-old female who showed features of IMPA, including acanthosis nigrans, hirsutism, acromegaly, and highly elevated insulin levels (23). To determine the etiology of these symptoms, the proband and her family members underwent exome sequencing, which identified 1108 potential deleterious variants. The list of deleterious variants was narrowed down based on gene set enrichment analysis of pathways relevant to insulin and IGF1 signaling, from which *FGFR1* (Val102Ile) and *KLB* (Ser9Tyr) were selected as potential candidates. Functional analysis of the *FGFR1* Val102Ile and *KLB* Ser9Tyr variants in L6 myoblasts showed strongly reduced FGF21-stimulated ERK phosphorylation, while FGF2-stimulated ERK phosphorylation was intact (23). How the Ser9Tyr missense variant affects KLB protein function is still obscure. As Ser9Tyr maps to the signal peptide of the protein, KLB localization may be disturbed. Thus, additional functional studies are needed to address the effect of Ser9Tyr on other aspects of the KLB protein, such as thermal stability, cell surface expression, or FGFR1 binding. In addition, a precise disease mechanism explaining how digenic variants in *FGFR1* and *KLB* cause IMPA is still lacking.

The discrepancy between observed and expected numbers of LoF or missense variants in the *KLB* gene and the absence of homozygous LoF carriers strongly indicate that this gene is protected against deleterious mutations. In addition, given that most of the *KLB* missense variants are not found in a homozygous state, the genetic contribution of KLB to disease will be mainly through heterozygous or oligogenic inheritance models. A potential pathogenic role of heterozygous *KLB* mutations is supported by various phenotypes displayed by heterozygous *Klb-*KO mice, including disturbed estrous cycle, blunted LH surge at the estrus stage, reduced pregnancy rate, reduced adiposity, and altered regulation of thermogenic gene expression (22, 34). However, more extensive phenotypic characterization of heterozygous *Klb*-KO mice or human cell systems is needed to address the full impact of *KLB* gene dosage. In addition, the generation of heterozygous *Fgfr*/*Klb* LoF mice could provide more insight into the potential deleterious effect of digenic *FGFR/KLB* variants. Moreover, due to the conflicting functional consequences of some *KLB* variants, the use of different functional assays, and the fact that the potential pathogenicity of the currently identified *KLB* missense variants is not uniformly predicted by several widely used gene variants effect prediction tools (**Table 2**), further studies addressing the functional consequence of *KLB* variants should incorporate previously identified variants and additional controls as recommended by The clinical Genome Resource Sequence Variant Interpretation Working group (35).

**Table 2:** Prediction of *KLB* variant pathogenicity through various gene variant interpretation tools.

KLB variant	SIFT	Poly-Phen	CADD	REVEL	MetaLR	Mutation Assessor
Ser9Tyr	Deleterious – low confidence	Possibly damaging	Likely benign	Likely benign	Tolerated	Low
Arg309Trp	Deleterious	Probably damaging	Likely benign	Likely benign	Tolerated	Medium
Arg309Gln	Tolerated	Benign	Likely benign	Likely benign	Tolerated	Low
Arg424Cys	Tolerated	Possibly damaging	Likely benign	Likely benign	Tolerated	Low
Ala574Thr	Tolerated	Possibly damaging	Likely benign	Likely benign	Tolerated	Low
Arg728Gln	Tolerated	Benign	Likely benign	Likely benign	Tolerated	Low
Lys815Glu	Deleterious	Possibly damaging	Likely benign	Likely benign	Tolerated	High
Leu1011Pro	Deleterious	Possibly damaging	Likely benign	Likely benign	Tolerated	Medium

SIFT, Sorting Intolerant From Tolerant tool; Poly-Phen, Polymorphism Phenotyping v2 tool; CADD, Combined Annotation Dependent Depletion tool; REVEL, Rare Exome Variant Ensemble Learner.

Identifying phenotypes that are modulated by KLB gives a deeper insight into human KLB biology. Some of these phenotypes may also be considered in clinical trials examining the safety and efficacy of KLB-targeting drugs. For example, the association of *KLB* variants with colonic transit may explain why the administration of KLB-targeting drugs is associated with gastrointestinal side effects. Whether these drugs also modulate other physiological systems potentially modulated by KLB, such as reproduction and alcohol intake, remains to be established. Furthermore, by altering FGF binding, specific *KLB* variants may also explain differences in drug response between individual patients.

### 2.2 Regulation of KLB expression

**Figure 1:**
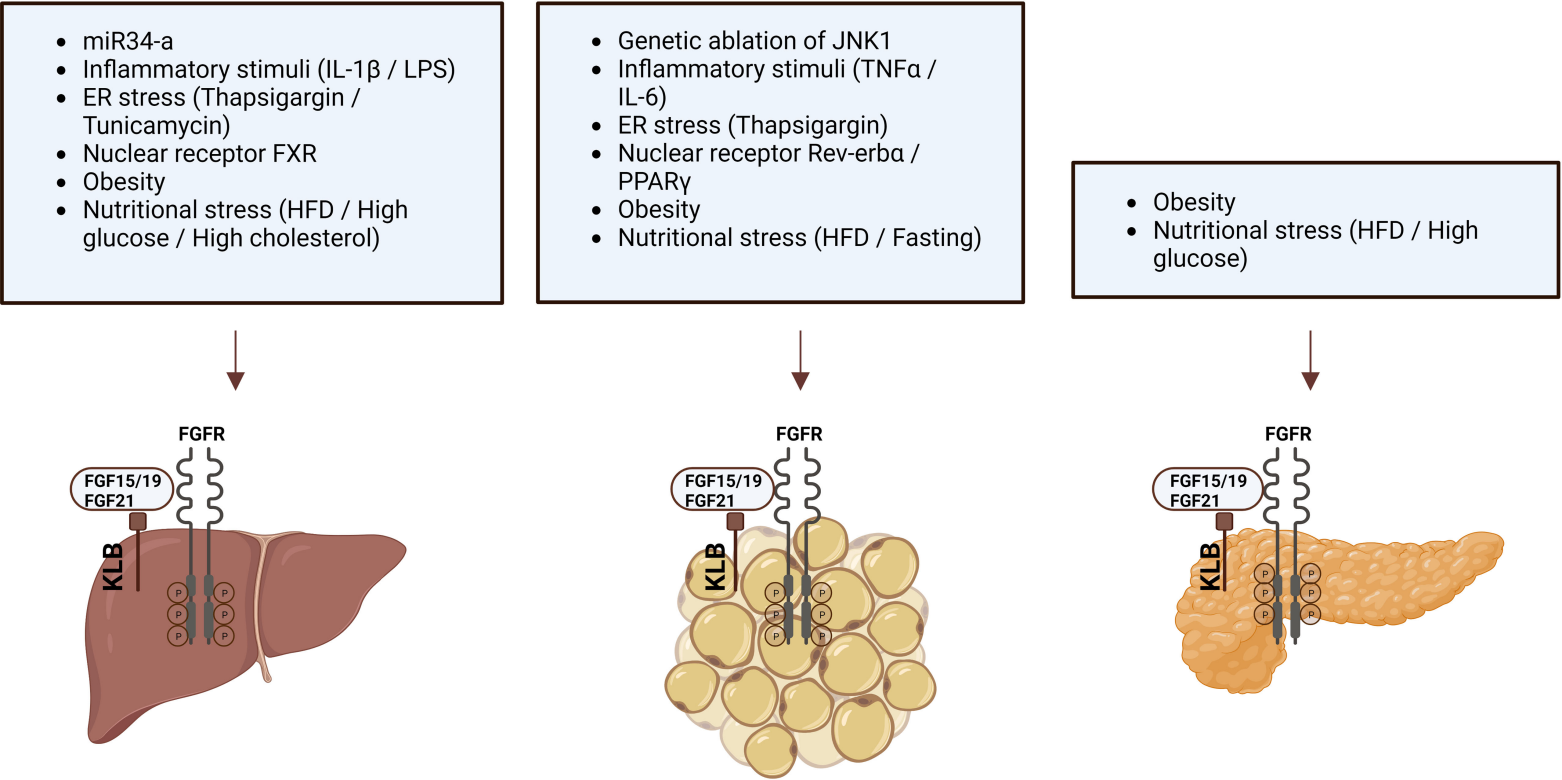
Pathophysiological factors that influence KLB expression in liver, adipose tissue, and pancreas.

Since FGF19/FGF21 activity depends on the presence of KLB, changes in KLB expression may alter the function of endogenous FGF19/FGF21 or the efficacy of FGF19/21-mimetics. Therefore, factors that change *KLB* mRNA expression or protein levels have been studied in multiple organisms and tissues (**Table 3**; **Figure 1**). Species in which KLB expression has been studied include mice, pigs, seal elephants, non-human primates, and humans. Tissues that were studied primarily included those expressing high levels of KLB, such as adipose tissue, pancreas, and liver. In most of these tissues, pathophysiological factors appear to reduce *KLB* mRNA expression or protein levels. In contrast, two studies reported an obesity-driven increase in KLB expression in the liver (42, 44). Unfortunately, not all studies measured mRNA and protein levels or performed extensive antibody validation. Pathophysiological factors that alter KLB expression can be grouped into three categories: obesity, fasting, and inflammation. As both obesity and fasting can also affect inflammation (50, 51), inflammatory and cellular stress pathways appear to play a key role in regulating KLB expression. Direct support for this idea comes from several studies that evaluated the effects of inflammatory- and endoplasmic reticulum (ER) stress on KLB expression in *in vitro* or *in vivo* models.

**Table 3:** Overview of studies reporting differential *KLB* mRNA expression or KLB protein levels.

Species	Condition	Tissue	mRNA/protein	Effect	Study
*Mus musculus*	Obesity	Adipose tissue	mRNA	↓	Fischer, 2010. (36)
*Mus musculus*	Obesity	Adipose tissue	mRNA	↓	Hale, 2012 (37).
*Mus musculus*	HFD	Liver	mRNA & protein	↓	Fu, 2012 (38).
*Mus musculus*	HFD	Adipose tissue	mRNA & protein	↓	Diaz-Delfin, 2012. (39)
*Mus musculus*	JNK^-/-^ mice	Adipose tissue	mRNA & protein	↑	Diaz-Delfin, 2012. (39)
*Mus musculus*	Genetic obesity (*db/db*)	Islets	mRNA & protein	↓	So, 2013 (40).
*Macaca Mulatta*	HFD	Pancreas	mRNA	↓	Nygaard, 2014. (41)
*Macaca Mulatta*	HFD	Adipose tissue	mRNA	↓	Nygaard, 2014. (41)
*Mus musculus*	HFD	Liver	Protein	↑	Dong, 2015 (42).
*Mus musculus*	Genetic obesity (*ob/ob*)	Liver	Protein	↑	Dong, 2015 (42).
*Homo sapiens*	NAFLD	Liver	Protein	↑	Dong, 2015 (42).
*Mus musculus*	LPS	Liver	mRNA & protein	↓	Zhao, 2015 (43).
*Mus musculus*	IL-1β	Liver	mRNA & protein	↓	Zhao, 2015 (43).
*Homo sapiens*	Obesity	Liver	mRNA	↑	Gallego-Escuredo, 2015 (44).
*Homo sapiens*	Obesity	Adipose tissue	mRNA & protein	↓	Gallego-Escuredo, 2015 (44).
*Mirounga* *angustirostris*	Fasting	Adipose tissue	mRNA	↓	Suzuki, 2015 (45).
*Mus musculus*	HFD	Adipose tissue	mRNA & protein	↓	Markan, 2017. (46)
*Homo sapiens*	Fibrosis	Liver	mRNA	↓	Lee, 2018 (47).
*Homo sapiens*	Fasting	Adipose tissue	mRNA	↓	Nygaard, 2018. (48)
*Homo sapiens*	NASH	Liver	Protein	↓	Dongiovanni, 2020 (20).
*Sus scrofa domesticus*	Fat, fructose and cholesterol diet	Liver	mRNA	↓	Cirera, 2020 (49).

JNK, c-Jun N-terminal kinase; NAFLD, non-alcoholic fatty liver disease; LPS, lipopolysaccharide; IL-1β, interleukin 1β; NASH, non-alcoholic steatohepatitis; HFD, high-fat diet.

Based on the idea that obesity is associated with a chronic pro-inflammatory state, Diaz-Delfin et al. analyzed the effect of inflammatory stimuli on the KLB/FGFR1 pathway in mouse 3T3-L1 adipocytes and showed that TNFα repressed Klb mRNA and protein expression (39). In addition, they demonstrated that the deletion of JNK1, which functions as a sensor of inflammatory status and cellular stress, increased Klb expression in WAT of mice (39). In hepatocytes, KLB expression appears less sensitive to TNFα or IL-6 stimulation (43, 47). Instead, treatment of the hepatoma cell lines Huh-7 and HepG2 or mouse liver with IL-1β strongly repressed KLB expression (43). IL-1β appears to regulate KLB expression at the transcriptional levels as the effect was blocked by actinomycin, an inhibitor of gene transcription, but not by cycloheximide, an inhibitor of protein synthesis (43). Moreover, IL-1β-driven repression of KLB was primarily mediated through the JNK and NF-κB pathways (43, 47).

Several studies have reported that ER stress modulates KLB expression. Thapsigargin, an activator of ER stress and stimulator of pro-inflammatory signals, strongly reduced Klb expression in 3T3-L1 adipocytes (39). In contrast, in hepatocytes, the ER stressors thapsigargin and tunicamycin increased KLB expression (42). Furthermore, the upregulation of KLB by tunicamycin was blocked after genetic inhibition of activating transcription factor 4 (ATF4), a transcription factor involved in the unfolded protein response (UPR) (42). These studies suggest that inflammatory stimuli and cellular stressors affect KLB expression in adipocytes and hepatocytes. A role for cellular stress in the regulation of KLB expression was also supported by the finding that treatment with high glucose (28 mmol/L) repressed *Klb* expression and FGF21 signaling in islets obtained from healthy mice (40). Thus, even though the transcriptional regulation of KLB is sensitive to cellular stress, the directionality of this appears to be controlled in a stimulus- and cell type-specific manner.

In addition to inflammation and cellular stress, several members of the nuclear receptor family, including Rev-erbα, BA-sensing transcription factor farnesoid X receptor (FXR), and the lipid sensor peroxisome proliferator-activated receptor gamma (PPARγ), have been identified as regulators of KLB expression in hepatocytes, adipocytes, and pancreatic islets. The role of Rev-erbα in controlling KLB expression was revealed after transcriptome analysis of Rev-erba-KO mice, showing that KLB mRNA and protein levels were strongly induced in epididymal WAT (eWAT) and inguinal WAT (iWAT) but not in liver, hypothalamus, and brown adipose tissue (BAT) (52). Immunoprecipitation followed by Chip-seq analysis revealed direct binding of Rev-erbα within the first intron of the *Klb* locus, showing that *Klb* is a direct target gene of Rev-erbα. PPARγ was similarly found to be a transcriptional regulator of KLB as it can bind the Rev-erbα binding sites in the *Klb* locus in eWAT and 3T3-L1 cells, and siRNA-mediated silencing of PPARγ in 3T3-L1 cells dramatically reduced *Klb* mRNA expression (52). Also, the PPARγ agonist rosiglitazone robustly stimulates KLB expression in adipose tissue, suggesting that PPARγ activation may enhance FGF21 sensitivity (39, 53). In addition, rosiglitazone counteracted the TNFα-, high-fat diet-, and high glucose-driven reduction in KLB expression in adipocytes, WAT, and pancreatic islets, respectively (39, 40). The third nuclear receptor found to regulate KLB expression was FXR, after previous studies demonstrated FXR binding peaks at the *Klb* locus in mouse liver (54). Additional studies revealed that the FXR agonist GW4064 stimulated FXR binding and RNA polymerase II occupancy at the *Klb* gene in mouse livers, consequently leading to increased KLB mRNA and protein levels. Conversely, in the livers of FXR KO mice, Klb mRNA and protein levels were decreased (55). Therefore, FXR protects the liver against high BA levels by inducing Klb expression, ultimately lowering *Cyp7a1* expression and BA synthesis.

Finally, regulation of KLB by small noncoding RNAs has also been demonstrated (38, 56). The 3’UTR of mouse and human *KLB* mRNA contains sequences that are partially complementary to *miR-34a*, the most highly upregulated microRNA (miRNA) in the liver of obese mice and patients with NAFLD or T2D (57, 58). Downregulation of *miR-341* was shown to increase Klb expression in mouse Hepa1c1c7 cells, while *miR-34a* overexpression decreased KLB levels in Hepa1c1c7 cells and mouse liver (38). In addition, direct binding of *miR-34a* to the 3’UTR of KLB was demonstrated. Normalization of *miR-34a* by antisense inhibition restored Klb levels in the liver of mice fed a HFD, which are typically characterized by reduced Klb expression (38, 56).

Although multiple factors that regulate KLB transcription have been identified, the functional consequences of altered KLB expression for FGF19/FGF21 activity remain controversial. Reduced KLB expression has been suggested to play a role in a concept called FGF21 resistance, a pathophysiological state in rodents and humans with obesity associated with increased plasma FGF21 levels, reduced KLB/FGFR expression, or a reduced response to exogenously administered FGF21 (46, 59–61). The functional implications of reduced KLB expression can be observed in heterozygous *Klb* mice, which show a 50% reduction in KLB protein levels, and display a variety of phenotypic changes, such as an altered LH surge, reduced adiposity, and altered thermogenesis (22, 34). Moreover, conditions that reduce KLB expression, including a loss of FXR, overexpression of *miR34-a*, or siRNA-mediated KLB downregulation, are associated with blunted FGF19 activity in hepatocytes (38, 55). Conversely, conditions that increase KLB expression, such as loss of Rev-erbα and overexpression of KLB in hepatocytes, enhanced FGF21-stimulated transcriptional activity in WAT and restored FGF19 activity in FXR-deficient hepatocytes, respectively (52, 55). Similarly, transgenic overexpression of *Klb* in WAT of mice was associated with enhanced sensitivity to both endogenous and exogenous FGF21 (61). In contrast to these reports, Hale et al. showed that although *Klb* expression was strongly decreased in *ob/ob* and DIO mice, the *in vivo* metabolic effects of exogenous FGF21 were largely preserved (37). In addition, Markan et al. demonstrated that transgenic overexpression of *Klb* in WAT of mice did not affect exogenous FGF21 sensitivity (46). Differences between mouse models can potentially explain the conflicting findings between these studies. Samms et al. used the *aP2* promotor to overexpress KLB in adipose tissue, which is also active in the brain, while Markan et al. used the adiponectin promotor, which is more specific for adipose tissue (46, 62). Therefore, some tissues may be more sensitive to altered KLB expression than others. Furthermore, the use of different functional readouts for measuring FGF19/FGF21 activity (e.g., ERK phosphorylation, FGF target gene expression, or metabolic effects) may have further contributed to discrepancies between these studies. Thus, while the transcription of the *KLB* gene appears to be sensitive to a wide range of pathophysiological factors, the consequences of altered KLB expression for endogenous or exogenous FGF19/FGF21 activity remain incompletely understood.

### 2.3 KLB protein structure

To obtain a better molecular understanding of the role of KLB in FGF19 and FGF21 signaling, structures of the free and ligand-bound extracellular region of human KLB protein were characterized using X-ray crystallography (63, 64). The KLB protein comprises 1044 amino acids and has a molecular weight of 120 kDa. The tertiary protein structure contains two tandem glycoside hydrolase-like domains, designated as D1 (or KL1, amino acid residues 53-507) and D2 (or KL2, amino acid residues 521-968) (63). Both D1 and D2 show substantial structural similarity to the human cytosolic B-glucosidase enzyme, demonstrating the evolutionary relationship of KLB to the glycoside hydrolase family-1 (GH1) enzymes. GH1 enzymes catalyze the hydrolysis of glycosidic bonds in carbohydrate molecules through two conserved glutamate residues in their active sites. In both D1 and D2 of KLB, one of these two conserved glutamate residues is replaced, suggesting that the glycoside hydrolase-like domains in KLB are not catalytically active (63). Another feature of KLB is a signal sequence (amino acid residue 1-53), of which the function is yet unknown.

Structural analysis of KLB bound to the C-terminal tail of FGF21 revealed the involvement of Pro186-Ser209 from FGF21 to an elongated interface spanning D1 and D2 of KLB. The binding of FGF21 to KLB did not appear to induce any conformational change in D1 or D2. In addition, two distinct binding sites in KLB interacting with two different regions of FGF21 were identified. The first site in D1 interacts with Pro186-Val197 of FGF21 largely through hydrophobic interactions. Site 2 in D2, which resembles the substrate-binding pocket normally occupied by glycosides in active GH-1 enzymes, interacted with residues 200-209 of FGF21. Interactions between the C-terminal part of the FGF21 molecule and the two KLB binding sides were validated by analyzing the functional consequences of a range of FGF21 and KLB variants in ligand-binding and cell stimulation experiments. These experiments showed that residues D192, P193, S204, S206, and Y207 in FGF21 could bind to KLB. In addition, mutations of relevant residues in D1 (V392, T431, and M435) and D2 (Y643, H646, E693, R696, R829, and R845) of KLB reduced FGF19- and FGF21-stimulated FGFR1 tyrosine phosphorylation (63, 64). Interestingly, some of these mutations also affected FGFR1 activation by FGF1 (63), raising the possibility that KLB shows some promiscuity towards other FGF members. Alternatively, KLB may also regulate FGFR signaling independent of its ligand-binding function, as suggested by its ability to control FGFR4 stability (65).

The structural analysis of ligand-bound KLB obtained with X-ray crystallography was replicated in another study using hydrogen deuterium exchange coupled to mass spectrometry (HDX-MS), which measures isotopic hydrogen exchange in protein backbone amides (66). As amides in the ligand-binding interface are protected from isotopic hydrogen exchange, this method allows for the sensitive determination of protein-protein interactions. HDX-MS revealed that the binding of FGF19 or FGF21 did not significantly alter the overall KLB structure, which is in line with structural data obtained with X-ray crystallography. Similarly, peptide regions in KLB involved in FGF19 and FGF21 binding mapped mainly to the D1 and D2 domains. Scanning mutagenesis followed by an FGF21 binding assay was used to assess these interactions in more detail, revealing that mutating residues Y434, M435, Y753, D852, and S858 strongly reduced FGF21 binding. These amino acids fall within the two FGF21-binding sites identified in KLB by X-ray crystallography. Using a similar approach, KLB bindings sites in FGF21 were also determined, revealing that residues 183-198 and 204-209 were the most strongly protected against isotopic hydrogen exchange, indicating that these two regions constitute the primary bindings site for KLB. These regions map to the C-terminus of FGF21 and overlap with regions previously identified using X-ray crystallography. Scanning mutagenesis followed by a KLB binding assay and Elk-1 luciferase reporter activity identified specific FGF21 residues involved in KLB binding, including D192, P193, L194, M196, and four residues near the distal part of the C-terminus (S204, P205, S206, and Y207). Similar to FGF21, two stretches of highly conserved amino acids in FGF19 (D198, P199, F200, L202, and four residues near the distal part of the C-terminus, including S211, P212, S213, and F214) strongly modulate KLB binding (66).

**Figure 2:**
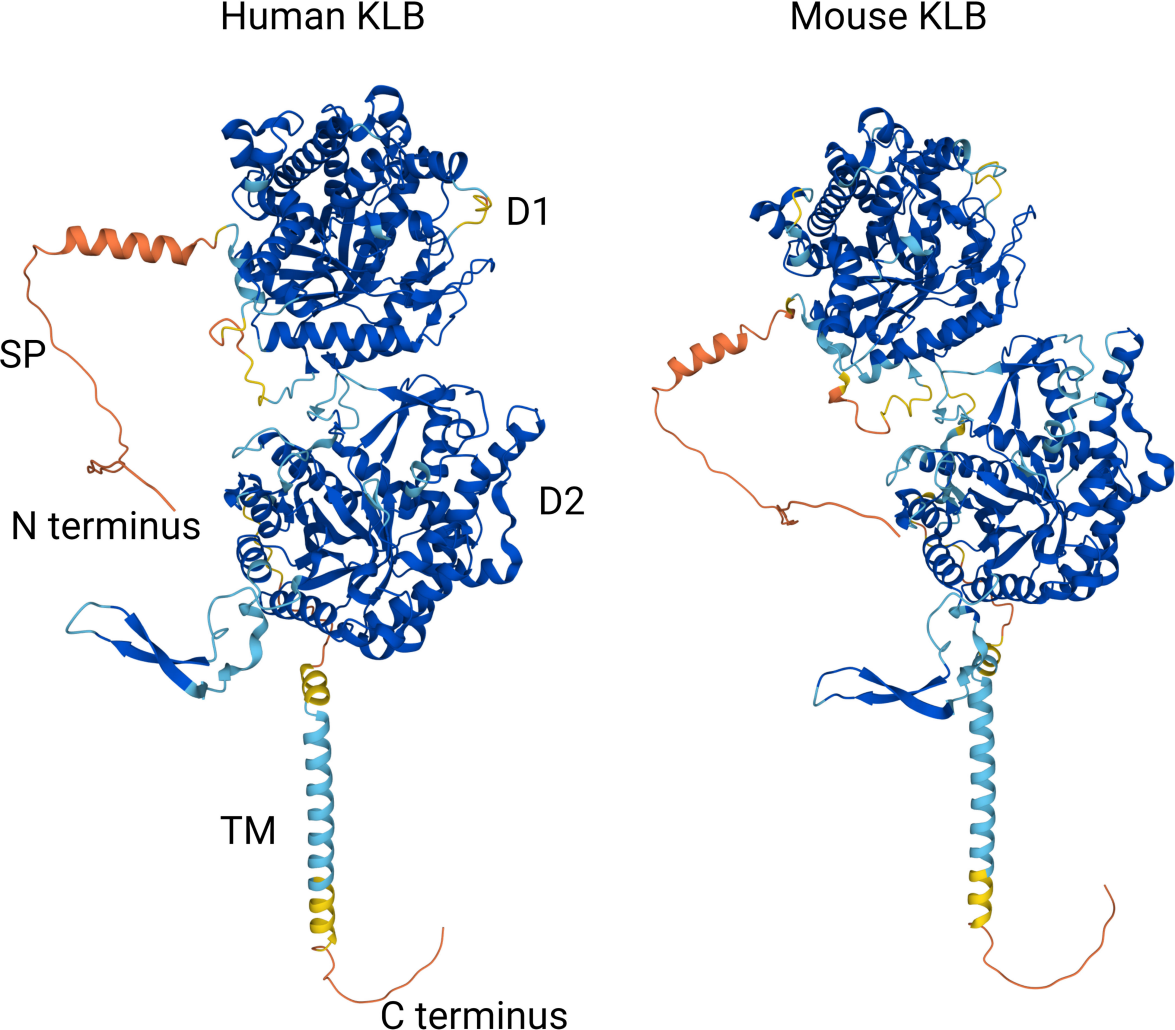
Comparison of Alphafold-based *in silico* predictions of the human and mouse KLB protein structures (67). Colors reflect model confidence (dark blue: very high (pLDDT >90); light blue: confident (90 < pLDDT > 70); yellow: low (70 > pLDDT > 50); orange: very low (pLDDT < 50). D1: domain 1 (residues 53-507); D2: domain 2 (residues 521-968); SP: signal peptide; TM: transmembrane region.

Based on these structural analyses of ligand-bound human KLB, a model has been proposed in which KLB primarily functions as a high-affinity receptor for FGF19 and FGF21 by binding to two conserved peptide sequences in their C-terminal tails. The binding of the globular core of FGF19 or FGF21 to FGFR then leads to the formation of the FGF-FGFR-KLB ternary complex and downstream tyrosine kinase activity. In addition, the strong structural similarity of D1 and D2 in KLB to glycoside hydrolases suggests that these enzymes have evolved to serve as a high-affinity cell surface receptor for endocrine FGFs. Finally, although the structure of the mouse KLB protein has not yet been elucidated experimentally, Alphafold-based *in silico* predictions show that the global structures of the mouse and human KLB proteins are highly similar despite their relatively low sequence homology (79%) (**Figure 2**) **(** 67).

### 2.4 Tissue distribution and subcellular localization of KLB

While FGFRs are ubiquitously expressed in the body, KLB is expressed more selectively, acting as the limiting factor determining in which tissues FGF15/19 and FGF21 are functionally active (61, 68). Although adipose tissue, liver, and pancreas are the central tissues known to express KLB, recent technological advancements, such as RNAscope *in situ* hybridization, droplet digital PCR, and single-cell RNA sequencing, have also identified KLB expression in other organs and cell types (69).

Initial efforts to determine *Klb* tissue distribution in embryonic and adult mice revealed that *Klb* transcripts became detectable from day 11 of gestation and expression levels increased further during development. Between gestational ages 10.5 and 19.5, *Klb* transcripts were mainly detected in the yolk sac, liver, gut epithelium, acinar cells of the pancreas, cervical BAT, and WAT. In adult mice, the highest *Klb* expression was detected in the liver and pancreas (5). Based on these initial observations, *Klb* transcripts appear to be expressed primarily in organs that control metabolic processes. Fon Tacer et al. established a comprehensive expression atlas of the FGF systems in adult mice using quantitative PCR in 39 different mouse tissues (70). In this study, the highest *Klb* expression was found in BAT, WAT, liver, gallbladder, colon, and pancreas, which is in line with previous studies and consistent with the well-known effects of FGF15/19 and FGF21 in these organs (70–72). In addition, *Klb* expression levels could also be detected in several other tissues, including the eye, adrenal gland, thyroid gland, stomach, small intestine, thymus, ovaries, testes, aorta, muscle, and skin, implying a potential role of KLB in many other homeostatic systems as well. For example, co-expression of *Klb* and *Fgfr4* in the adrenal tissue and the aorta suggests their involvement in FGF15/19- or FGF21-mediated effects on steroidogenesis and blood vessel physiology, respectively. In line with this, FGF21-mediated protective metabolic effects on the heart have been reported (73–77). However, it is unknown whether these effects are dependent or independent of KLB. Unsupervised hierarchical clustering based on mRNA expression demonstrated that *Klb* is primarily enriched in the gastrointestinal (GI) and reproductive system and most strongly co-expressed with *Fgfr4* (65).

Although *Klb* is not ubiquitously expressed in the rodent central nervous system (CNS), various observations suggest that FGF21 acts centrally (70). Using anatomically guided laser-capture microdissection followed by quantitative PCR, *Klb* expression was detected in the suprachiasmatic nucleus (SCN), hippocampus, and several midbrain and hindbrain nuclei (78). Expression of *Klb* in these brain regions has been shown to mediate many of the endogenous and pharmacological effects of FGF21, such as the suppression of physical activity, modulation of circadian behaviour, and the activation of sympathetic nerve activity (79). Through automated RNAscope *in situ* hybridization and droplet digital PCR technology, Hultman et al. were able to define central *Klb* expression in mice further. Besides the SCN, *Klb* transcripts were also found in the reticular thalamus, the medial vestibular nucleus and the medial trigeminal neurons of the midbrain, and the hypoglossal nucleus of the hindbrain (69). A more recent study also identified *Klb* expression in hypothalamic neurons and amygdalar nuclei (80).

**Figure 3:**
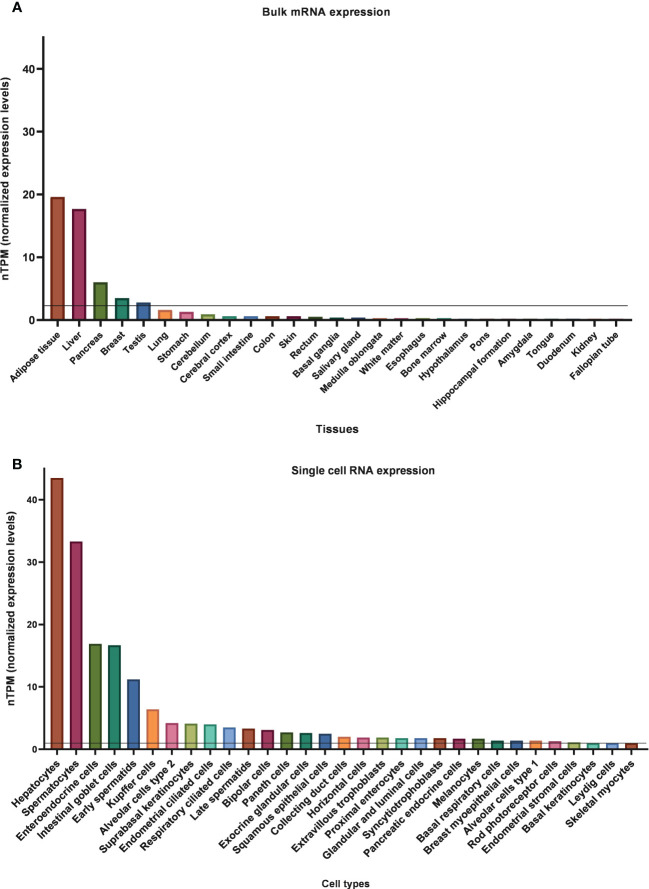
Normalized bulk tissue **(A)** and single-cell **(B)** KLB mRNA expression data obtain from HPA (81, 82). 1.0 nTPM was used as a cut-off for detection.

*KLB* mRNA expression in human tissues is easily accessible and comparable due to many publicly available databases. The consensus dataset of the Human Protein Atlas (HPA), which reports expression levels of 55 human tissues by combining HPA and GTEx transcriptomics datasets, indicates that *KLB* is predominantly present in adipose tissue, liver, and pancreas (**Figure 3**). Using 1 nTPM as a cut-off for detection, *KLB* transcripts can also be detected in breast tissue, testis, lung, and stomach (81). In contrast to rodents, *KLB* does not appear to be expressed in the human brain. Human studies using automated RNAscope *in situ* hybridization and droplet digital PCR technology revealed that *KLB-*positive cells were only scarcely found in the human mid- and hindbrain, showing a discrepancy in *KLB* expression between rodents and humans (69). Single-cell RNA sequencing revealed that *KLB* transcripts are enhanced in hepatocytes (liver), spermatocytes (testis), alveolar cells type 2 (lungs), enteroendocrine cells (colon), and glandular intestinal cells (small intestine and rectum) (82). The presence of *KLB* transcripts in these tissues and cell types suggests that it also has other functions beyond metabolic regulation.

Although KLB is traditionally viewed as an FGF coreceptor located in the plasma membrane, relatively few studies have addressed its subcellular localization. Zweers et al. performed immunohistochemistry on sections of the gall bladder, small intestine, colon, and liver obtained from patients undergoing abdominal surgery. In line with publicly available transcriptomic datasets, KLB and FGFR4 showed the highest expression in the liver. Within the cell, KLB was detected in the cytoplasm and the plasma membrane (71, 83). While KLB was mainly present in the cytoplasm, a specific granular staining pattern was observed for FGFR4 in the gall bladder, which was attributed to the apical plasma membrane and ER or Golgi structures, indicating a possible interaction of KLB with the FGFR4 in the ER or Golgi. In line with this observation, Triantis et al. reported that KLB is resident in the ER (65). Computational protein analysis using the PSORTII interface predicted a 45% chance of KLB localization in the ER compared to a 33% chance for Golgi and a 22% chance for the plasma membrane. Additional immunofluorescence studies in kidney fibroblast-like COS-7 cells transfected with KLB demonstrated that KLB colocalized with the pEGFP-ER marker but not with the pEYFP-Golgi marker (65). Interestingly, this study also showed that KLB engaged in the glycosylation of FGFR4 in the ER. Based on these observations, KLB was suggested to interact with the inactive core glycoform of FGFR4 and direct it toward the proteasome. As a result, the active terminal glycoform reaches the plasma membrane and can be activated by FGF19 (65). These results fuel the hypothesis that KLB could be involved in processing receptor complex molecules or proteasomal degradation aside from its conventional role as a coreceptor (65). Even though Triantis et al. detected the presence and interaction of KLB with FGFR4 in the ER, it remains obscure whether KLB is directly involved in proteasomal degradation and, if so, which mechanism of proteasomal degradation removes inactive components (65). Further studies are required to establish the exact subcellular location of KLB within the cell and its impact on the function of other organelles.

## 3 Functions of KLB

### 3.1 Liver and gallbladder

The first and best-described function of KLB concerns its role in the physiologic regulation of BA synthesis (84). Postprandial release of bile acids activates FXR in the ileal enterocytes, leading to the expression and secretion of FGF15/19. Parallel to intestinal FXR activation, bile acids also activate hepatic FXR, which increases KLB expression in hepatocytes (55). Subsequently, secreted FGF15/19 induces KLB/FGFR4 complex formation in the liver, resulting in the transcriptional repression of *CYP7A1* (85). As *CYP7A1* encodes the rate-limiting enzyme in BA synthesis, FXR-mediated induction of ileal FGF15/19 and hepatic KLB controls the negative feedback regulation of BA synthesis (55, 86, 87). Evidence for the role of KLB in BA metabolism was provided by *Klb*-deficient mice, which were characterized by increased *Cyp7a1* expression, strongly elevated synthesis and excretion of bile acids, and an altered BA composition (84, 85, 88). Hepatocyte-specific transgenic overexpression of KLB in *Klb*-deficient mice restored *Cyp7a1* expression, BA pool size, and BA excretion (88). Together, these findings identified KLB as a suppressor of BA synthesis through negative regulation of CYP7A1 (85, 86, 89, 90). In humans, targeting KLB using FGF19-mimetics strongly reduced serum 7a-hydroxy-4-choleston-3-one (C4) levels, a marker of hepatic CYP7A1 activity, showing functional conservation of KLB in mice and humans (91). While suppression of BA synthesis plays a central role in the protective effects of FGF19-mimetics, this is less clear for FGF21-mimetics.

Since bile acids can regulate lipid and carbohydrate metabolism, it is no surprise that KLB is also involved in these processes. *Klb* deficiency in mice causes dysregulated lipid homeostasis, characterized by lower plasma triglycerides and a tendency towards lower plasma cholesterol, which was reversed upon hepatocyte-specific restoration of KLB (85, 88). Also, mice deficient in *Klb* show an altered carbohydrate metabolism, as reflected by decreased hepatic glycogen levels and increased hepatic glucokinase expression (85). As both processes are pivotal in glycolysis, KLB is suggested to play a role in glucose turnover. As a result of the altered hepatic metabolism, *Klb*-deficient mice develop a pathological phenotype characterized by the presence of hepatic lipid accumulation, increased expression of pro-inflammatory cytokines and macrophage markers, and increased plasma alanine aminotransferase (ALT) and aspartate aminotransferase (AST) levels (85).

Aside from its role in the liver, KLB also appears to regulate gallbladder function. *Klb*-deficient mice fed a lithogenic diet had a smaller gallbladder and showed increased secretion of BA into bile, revealing a protective effect of KLB against gallstone formation (70, 85, 90). Reasons suggested for this protective effect are enhanced gallbladder motility or a more hydrophilic and frequent BA pool cycling (90). Interestingly, in humans, the highest expression of FGF19 is observed in epithelial cells of the gallbladder, which is up to 400-fold higher than in the ileum (71, 92). Also, FGF19 protein levels in bile collected from the gallbladder are roughly ten times higher than FGF19 levels in circulation (21.9 *vs*. 0.22 ng/mL), suggesting active secretion of FGF19 into bile (71). As KLB is expressed on the apical side of epithelial cells of the gallbladder, it may, in combination with FGF19, modulate human gallbladder function in an autocrine or paracrine fashion (71). It is unknown whether pharmacologically administered KLB-targeting drugs can reach the epithelial cells lining the gallbladder lumen and impact their function.

### 3.2 Adipose tissue

The identification of FGF21 as a hormone that stimulates glucose uptake in adipocytes and the strong increase of KLB during adipocyte differentiation suggested that adipose tissue is a central player in the metabolic effects of FGF21 (93). Initially, studies that examined the role of adipose tissue in the metabolic effects of FGF21 were performed in mice with *aP2*-Cre promotor-driven KLB deletion. However, as this promoter is also active in non-adipose tissue, such as the CNS, these studies did not provide definitive proof that adipose is a direct target organ for FGF21 (68, 94). Later, indirect evidence for the role of adipose tissue in controlling the metabolic effect of FGF21 was provided by studies showing that FGF21 administration stimulates the expression and secretion of adiponectin, an insulin-sensitizing hormone that is specifically produced by the adipose tissue (95, 96). In these studies, the ability of FGF21 to lower blood glucose levels and improve insulin sensitivity in diet-induced obesity (DIO) mice was dependent on adiponectin secretion (96) since an adipose-specific deficiency of Klb or adiponectin in these mice eliminated the FGF21-mediated metabolic improvements (84, 95–97). Direct evidence of the role of adipose tissue in FGF21 activity was provided in lipodystrophic mice, which are refractory to the beneficial metabolic effects of FGF21 (98). Transplantation of healthy WAT into lipodystrophic mice could completely restore FGF21 activity (46). Additional evidence was obtained from mice with an adiponectin-Cre promotor-driven deletion of Klb, which is selective for the adipose tissue (97). This study showed that the acute insulin-sensitizing effects of FGF21 are driven by targeting KLB in adipose tissue, whereas most other metabolic effects originate from targeting central KLB. Earlier findings showing that FGF21-mediated metabolic effects are dependent on adiponectin could not be replicated (97). Interestingly, FGF21 is also able to target the adipose tissue independently of KLB, even though the underlying mechanism remains incompletely understood (89). Although the role of KLB expression in adipose tissue and downstream adiponectin secretion in the effects of FGF21 remain controversial, FGF21-based drugs consistently increase plasma adiponectin levels in humans and non-human primates (99), indicating that FGF21 also targets the adipose tissue in these species.

While FGF15/19 binds preferentially to FGFR4, it can also act through FGFR1c that is predominantly present in the white adipose tissue (100). Although FGF19 is able to increase EGR1 transcriptional activity in the adipose tissue *in vivo*, the metabolic effects of FGF19 do not appear to involve KLB expression (84, 89). Recently, an association was found between circulating FGF19 levels and UCP1 expression in subcutaneous white adipose tissue of obese individuals (101). This association was investigated further using mice overexpressing FGF15, revealing the induction of browning as shown by presence of beige cells and increased UCP1 protein levels. Similarly, FGF19 was found to promote energy expenditure in BAT through upregulation of PGC-1α and UCP1 expression (102). In the absence of FGF15, mice displayed impaired browning and reduced BAT activity. Therefore, FGF15/19 could potentially play a role in adipose tissue plasticity. Similar results were found for FGF21, showing that these endocrine FGFs are involved in the thermogenic adaptation to cold in the WAT and BAT (103). However, further research needs to establish whether these effects occur dependently or independently of KLB.

### 3.3 Brain

While KLB is mainly expressed in peripheral metabolic tissues like the liver, adipose, and pancreas, central Klb expression in rodents was anticipated since FGF21 could cross the blood-brain barrier and intracerebroventricular injection of FGF21 increased metabolic rate and insulin sensitivity in rats (78, 104, 105). Indeed, *Klb* transcripts were detected in the SCN of the hypothalamus, the area postrema, the nucleus of the solitary tract, and the dorsal vagal complex (30, 68, 80). Deletion of *Klb* in the hypothalamus and hindbrain revealed that FGF21 acts centrally to increase plasma levels of ketone bodies and glucocorticoids, suppress growth, and modulate circadian behavior (78, 106). In addition, FGF21 and KLB have been identified as critical players in the neuroendocrine control of female reproduction, in which FGF21 acts as a starvation signal to reduce female fertility (106). Overexpression of FGF21 affects female reproduction by inducing infertility due to delayed onset of puberty, a lower incidence of ovulation, and diminished LH levels. Mechanistically, FGF21 acts on the SCN to suppress vasopressin-kisspeptin signaling and inhibit LH release, revealing a neuroendocrine axis that controls female reproduction (106). A role for FGF21 and KLB in controlling reproduction is also supported by the observation that *Klb*-KO mice have impaired estrous cycles, diminished LH levels, and impaired fertility (22, 107). In addition, it has been shown that the metabolic adaptations that follow dietary protein restriction, including altered macronutrient preference, energy expenditure, growth, and insulin sensitivity, are driven by central FGF21/KLB signaling (108). In an attempt to identify the neural substrates through which FGF21 regulates metabolic adaptations in response to protein restriction, Flippo et al. (2020) found that protein restriction-driven protection against weight gain was specifically controlled by KLB in glutaminergic neurons, and not by KLB in GABAergic neurons (109). In contrast, protein restriction-driven enhancement of insulin sensitivity was independent of glutamatergic KLB signaling (108, 109). In addition, this study showed that the effect of FGF21 on protein preference is secondary to the suppression of simple sugar consumption (109). Furthermore, FGF21 is able to suppress alcohol consumption in rodents and non-human primates by targeting KLB-expressing neurons in the basolateral amygdala and counteract alcohol intoxication by activating noradrenergic neurons in the locus coeruleus (32, 110). Finally, it is becoming increasingly apparent that many beneficial metabolic effects of recombinant FGF19/FGF21 and FGFR1/KLB-targeting antibodies, such as increased energy expenditure, body weight loss, and secondary effects on glucose levels, are driven by targeting KLB in the brain. Targeting central KLB leads to subsequent activation of neuronal pathways that enhance sympathetic nervous activity in the BAT (79, 84). The ability of FGF21 to increase energy expenditure and promote weight loss appears to be controlled specifically by KLB-expressing glutamatergic neurons, but also involves complex interactions with central leptin signaling (111). In contrast, the acute insulin-sensitizing effects of FGF21 are retained in mice with KLB-deficient glutamatergic neurons (111). However, as *KLB* transcripts are not clearly detected in the adult human brain, it remains unclear if FGF19/FGF21 can act centrally in humans.

### 3.4 Pancreas

KLB has been shown to be involved in both the exocrine and endocrine functions of the pancreas (112). The exocrine pancreas is made up of ductal structures and acinar cells that produce and secrete digestive enzymes. As pancreatic acinar cells synthesize and secrete considerable amounts of proteins, their ability to maintain proteostasis to prevent misfolding and ER stress is vital. A potential role for KLB in pancreatic acinar cells was anticipated as they have been shown to express FGF21 in response to feeding or ER stress (92, 112). Indeed, studies using FGF21 treatment confirmed that FGF21 acts directly on pancreatic acinar cells to stimulate digestive enzyme secretion and pancreatic juice flow, as these effects were lost upon the knock-out of *Klb* (112). By stimulating digestive enzyme secretion, FGF21/KLB appear to reduce ER stress and pancreatic proteotoxicity (112). Whether KLB also modulates pancreatic acinar cell function in humans is currently unclear. However, a direct comparison of the different pancreatic cell types in humans shows that *KLB* is most highly expressed in exocrine cells (92).

Although *KLB* expression is lower in the endocrine pancreas compared to the exocrine pancreas, FGF21-dependent and -independent effects on islet function have been elucidated. The ability of FGF21 to suppress glucagon secretion from primary rat islets provided the first indication that KLB can affect islet function (113). In follow-up studies, FGF21 increased insulin content and secretion from primary islets of diabetic rodents, and increased β-cell mass and function (114, 115). FGF21 also protects β-cells from glucolipotoxicity and apoptosis by activating ERK and Akt signaling (115). While FGF21 could increase the insulin content and number of islets, there was no effect of FGF21 on islet cell proliferation. In contrast to these *in vitro* studies, long-term administration of FGF21 in *db/db* mice did not affect β-cell function and mass. KLB was also found to affect pancreatic homeostasis independently of FGF21. Geng et al. demonstrated that KLB could fine-tune glucose-stimulated insulin secretion (GSIS) via modulation of glycolysis in pancreatic β-cells independently of FGF21 (116). A β-cell-specific deficiency of KLB in mice resulted in impaired GSIS and glycolytic flux in islets, which could recover to normal upon restoration of KLB expression. Mechanistically, KLB was shown to interact with and stabilize cytokine receptor unit GP130 by preventing its degradation, resulting in STAT3-HIF1a signaling, which consequently activates glycolytic genes and promotes GSIS (116). Based on these observations, KLB was suggested as a protective factor against β-cell dysfunction and T2D development.

### 3.5 Intestine

In intestinal epithelial cells, KLB has been shown to positively regulate tight junction plasticity and maintain intestinal barrier integrity (117). This function was revealed when Klb overexpression was found to protect mice from hepatic steatosis and inflammation. KLB could maintain the intestinal epithelial barrier by forming tight junction complexes in enterocytes and preventing the endocytic degradation of these complexes (117). Ethanol was found to reduce KLB expression in enterocytes, thereby contributing to a disrupted intestinal barrier and leakage of unfavourable substances into the bloodstream. Upregulation of KLB in enterocytes was able to protect and restore this alcohol-induced dysfunctional intestinal barrier, supporting the hypothesis that KLB is essential for supporting intestinal barrier integrity (117). Besides regulating barrier integrity, a complex of KLB with FGF15/19 can regulate BA circulation through the apical sodium-BA transporter (ASBT) in the ileum. Intraperitoneal administration of FGF19 decreased ASBT protein in the ileum, whereas silencing of FGF15 or KLB in mice increased ASBT activity (72). In this way, KLB appears to be involved in regulating BA resorption in the intestine (72).

**Figure 4:**
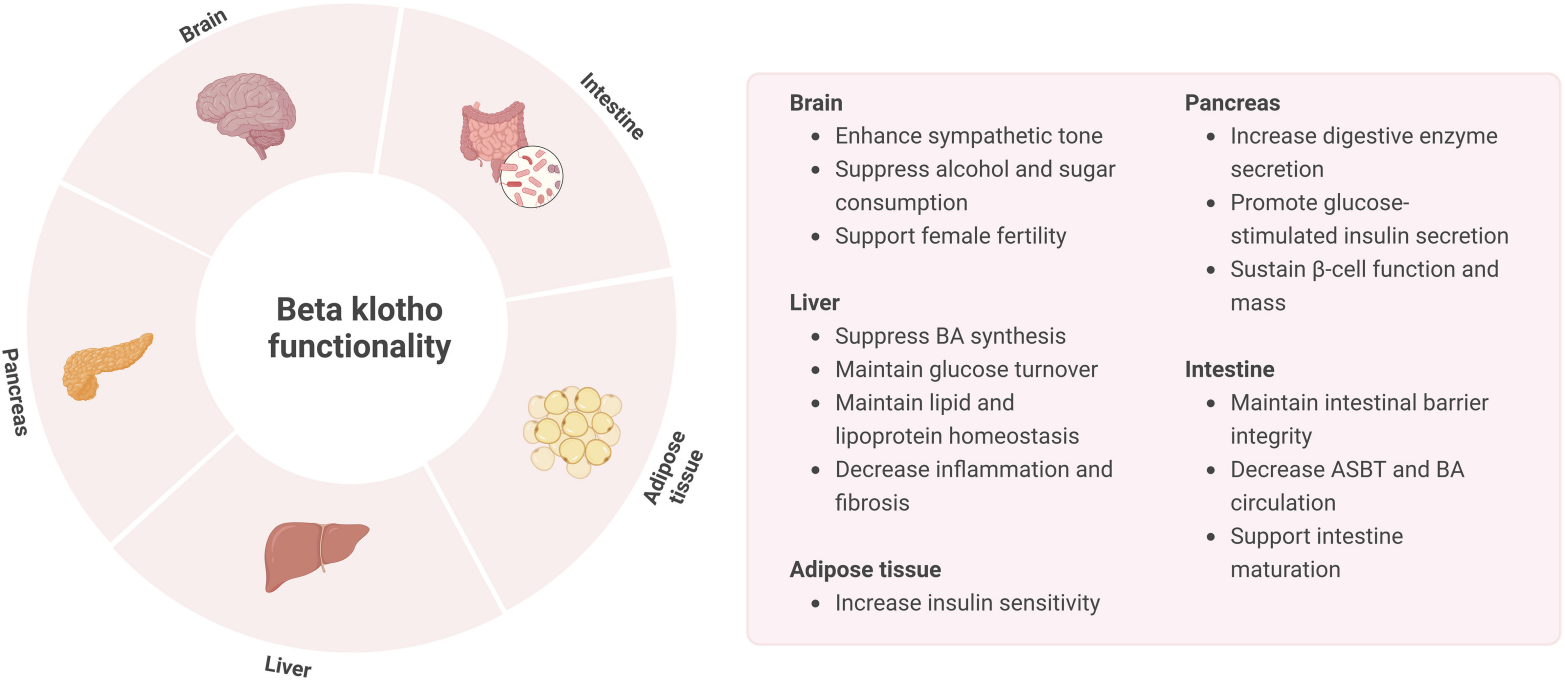
Overview of KLB functionality in the brain, liver, pancreas, intestine, and adipose tissue.

While FGF19 is mostly associated with BA metabolism in the liver and FGF21 with glucose and lipid metabolism in the adipose tissue, these endocrine factors also have overlapping actions. As such, FGF21 is able to regulate intestinal structure and function as well. Studies in mice have demonstrated that FGF21 treatment is able to decrease BA pool size, change gut microbiome composition and restore intestinal structure, thereby reducing NAFLD (118, 119). A recent study revealed that in neonatal FGF21-KO mice fed by FGF21-KO and WT lactating dams FGF21 in breast milk is able to induce intestinal hormones and digestive enzymes, lactase activity, and lactose absorption. These effects presumably act through KLB, which is highly expressed in the small intestine of neonates but not adults. Thereby, FGF21 was found to play a major role in the maturation and shaping of intestine development, possibly through KLB (120). However, the relevance of KLB in adults regarding intestinal function remains to be further investigated. An overview of KLB functionality is provided in **Figure 4**.

## 4 KLB-targeting drugs in clinical studies

Pharmacological targeting of the FGFR-KLB system dramatically improves metabolic disturbances in rodent models of obesity, T2D, and NAFLD, observations that paved the way for more advanced drug development. Meanwhile, pharmaceutical companies have designed and tested a multitude of FGF19- and FGF21-mimetics, recognizing KLB as a promising drug target for treating various metabolic diseases in humans. Although pharmacokinetic and pharmacodynamic properties of these drugs differ substantially, they all can target KLB. In this last section, we will highlight recent developments regarding the safety and efficacy of KLB-targeting drugs in clinical trials (**Table 4**). However, we recognize that the safety and efficacy of KLB-targeting drugs have also been extensively reviewed elsewhere (99, 133).

**Table 4:** KLB targeting drugs in clinical studies.

Drug name	Design	Company	Clinical trials	Treatment	References
NGM282	FGF19 analogue	NGM Biopharmaceuticals	NCT02135536 NCT05130047	NASH, PBC, PSC	(121–123)
LY2405319	FGF21 analogue	Lilly	ND	T2D	(124)
PF-05231023	FGF21 analogue	Pfizer	ND	T2D	(31, 125)
BMS-986036 (Pegbelfermin)	PEGylated FGF21 analogue	Bristol-Myers Squibb	NCT03486899 NCT03486912	NASH	(126–128)
AKR-001 (Efruxifermin)	Fc-FGF21 analogue	Akero Therapeutics	NCT04767529 NCT05039450	T2D, NASH	(129, 130)
BFKB8488A (bFKB1)	β-Klotho-dependent FGFR1c agonist	Genentech	NTC04171765	NASH	(131)
MK-3655 (NGM313)	FGFR1c/β-Klotho complex agonist	Merck Sharp & Dohme	NTC04583423	NASH	(132)

NASH, non-alcoholic steatohepatitis; PBC, primary biliary cholangitis; PSC, primary sclerosing cholangitis; T2D, type 2 diabetes; ND, no data available.

### 4.1 FGF19 analogue NGM282

Pharmacological administration of FGF19 improves metabolic disorders such as T2D and NAFLD. NGM282 (also called M70 or Aldafermin) is an FGF19 mimetic designed to induce FGFR1c and FGFR4 signaling without activating pathways involved in hepatocellular proliferation (122). NGM282 retained full metabolic activity, and preclinical studies showed overall metabolic improvement in animal models of NASH, including amelioration of bile acid toxicity, decreased hepatic inflammation and fibrosis, a reduction in hepatic lipid content, and improvement of insulin sensitivity (122, 123). NGM282 entered clinical trials with promising initial results. Twelve weeks of NGM282 treatment diminished liver fat content (-67-74% in 3 mg groups), improved liver damage markers and histological features of NASH (-1.9 NAS score in 3 mg group) in NASH patients in two phase 2 clinical trials (134, 135).

Recently, another phase 2 clinical trial was conducted to investigate the efficacy and safety of more prolonged treatment (i.e., 24 weeks) with NGM282 in biopsy-proven NASH patients (121). Longer NGM282 treatment decreased plasma C4 levels, liver damage markers ALT and AST, and liver fibrosis biomarker Pro-C3 (121). Histological outcome measures also improved in NGM282-treated patients with improvements in fibrosis and NAFLD activity score (NAS)(62%), and NASH resolution (121). One of the safety concerns of NGM282 treatment is the increase in plasma low-density lipoprotein (LDL) cholesterol, which can be managed by using rosuvastatin (134, 136).

As NGM282 treatment results in a marked reduction of bile acid levels, other clinical trials have studied the effect of NGM282 in patients with primary biliary cholangitis (PBC) and primary sclerosing cholangitis (PSC), both chronic liver diseases concerning bile acid-induced liver injury. NGM282 treatment in these patients reduced bile acids, plasma C4 levels, and various liver damage and fibrosis markers (137, 138). Currently, clinical trials using NGM282 focus on patients with PBC and chronic diarrhea due to bile acid malabsorption (ClinicalTrials.gov Identifiers: NCT02135536 and NCT05130047).

### 4.2 FGF21 analogues

Although FGF21 does not have mitogenic properties, the native protein is unsuitable for clinical use due to its poor pharmacokinetic properties. Therefore, several FGF21-based drugs with higher potency and pharmacokinetics were developed and tested in clinical trials. FGF21 analog LY2405319 (Lilly), the first to enter a phase 1 clinical trial, showed promising metabolic outcomes in preclinical studies in mice and monkeys on features of T2D (124, 139, 140). LY2405319 treatment of patients with obesity and T2DM for 28 days improved dyslipidemia, lowered body weight, reduced insulin levels, and increased adiponectin levels but did not result in the expected glucose-lowering effects (124). Another effort to make a long-lasting FGF21 mimetic resulted in PF-05231023 (Pfizer), which is in phase 1 clinical trial in obese patients with T2D, showing less improvement of dyslipidemia and lower body weight, but again did not lead to glucose lowering (31, 125). Furthermore, side effects included changes in bone turnover markers and blood pressure (125). Currently, no ongoing clinical trials are using LY2405319 and PF-05231023 (ClinicalTrials.gov).

Polyethylene glycol (PEG)-conjugated FGF21 variants were created to increase half-life and duration of action. Pegbelfermin (also called BMS-986036, Bristol-Myers Squibb) has made it to phase 2 clinical trials with promising results. Pegbelfermin treatment for 12 weeks improved dyslipidemia and insulin sensitivity, increased adiponectin levels, and decreased ALT, AST, and Pro-C3 levels in obese patients with T2D (126). Pegbelfermin treatment of patients with NASH was found to be well tolerated and resulted in a decrease in the hepatic fat fraction (5-7% in treated groups with 30% in >50% of patients) and improvement in liver stiffness and biomarkers of liver damage and fibrosis (128). Pegbelfermin entered the phase 2b FALCON studies (FALCON 1 and 2, ClinicalTrials.gov Identifiers: NCT03486899 and NCT03486912), in which patients with NASH, characterized by stage 3 liver fibrosis or compensated cirrhosis, received Pegbelfermin treatment for 24 and 48 weeks, respectively (127). Currently, the FALCON studies have been completed, and the results remain to be published.

Another promising FGF21-based drug is AKR-001 (Efruxifermin, Akero Therapeutics), a long-acting Fc-FGF21 fusion protein with increased binding affinity to KLB (141). In phase 1 clinical trial (ClinicalTrials.gov Identifier: NCT01856881) in patients with T2D, four weeks of AKR-001 treatment resulted in a reduced atherogenic lipid profile and improved insulin sensitivity with reduced plasma glucose, insulin, and HOMA-IR (129). In a phase, 2a clinical trial (ClinicalTrials.gov Identifier: NCT03976401), 16-week AKR-001 treatment in NASH patients caused a reduction of the hepatic fat fraction (10.3% - 14.1%), NASH resolution without worsening of fibrosis, and reduction in enhanced liver fibrosis (ELF) scores (130). Currently, AKR-001 is in phase 2b clinical trials for treating NASH in non-cirrhotic and compensated cirrhotic patients (ClinicalTrials.gov Identifiers: NCT04767529 and NCT05039450).

In summary, clinical trials using FGF21 mimetics generated disappointing results on glycemic control, making it unlikely that FGF21 mimetics will serve as a stand-alone treatment for T2D. On the other hand, the benefits of FGF21 therapeutics include improvement of the atherogenic lipid profile, hepatic fat fraction, and markers of liver damage and fibrosis, making these compounds promising for treatment strategies in NAFLD and NASH patients.

### 4.3 Direct targeting of the FGFR1/KLB complex

About a decade ago, bi-specific anti-FGFR1/KLB antibodies became of interest as an alternative approach for FGF21 mimetics to avoid global FGFR activation and to specifically activate FGFR1 signaling in a KLB-dependent manner. mimAb1 was one of the first identified human monoclonal antibodies that activated the FGFR1c/KLB complex (142). Effects of mimAb1 were tested in obese cynomolgus monkeys on a four-week treatment, which resulted in weight loss, decreased fasting and fed plasma insulin levels, and improved insulin sensitivity (142). In addition, an FGFR1c/KLB bispecific Avimer fused to human serum albumin called C3201-HSE was generated, which also induced FGF21-like effects in obese cynomolgus monkeys, among which decreased plasma insulin and triglyceride levels (143). A more extensive metabolic study using FGFR1c/KLB antibodies was performed using bFKB1, another selective KLB-dependent FGFR1c agonist (144). As the bFKB1 epitope differs from the binding site for FGF21, it does not affect endogenous FGF21 signaling. Like FGF21 mimetics, bFKB1 induced weight loss, lowered plasma glucose levels and hepatic triglycerides, increased energy expenditure, and induced BAT thermogenesis in obese mice (144). Follow-up studies demonstrated that bFKB1 stimulates BAT thermogenesis in a Ucp1-independent manner in mice and may act via the nervous system, similar to FGF21 (145). The promising effects of this drug, renamed BFKB8488A (Genentech), led to further studies in nonhuman primates and humans. A single administration of BFKB8488A to obese cynomolgus monkeys led to dose-dependent weight loss, reducing fat mass and food intake (146). An attempt has been made to overcome the distance between the two arms of bispecific monoclonal antibodies by creating a biparatopic molecule that could better mimic natural ligands. Using this approach, two different epitopes can be targeted with both close and distant proximity between the two antigen-binding sites. The biparatopic molecule targeting the FGFR1c/KLB complex, called IgG-VH1+VH2, has shown FGF21-mimicking effects *in vitro* but has not yet been further investigated in preclinical studies (147).

### 4.4 BFKB8488A in clinical trials

BFKB8488A is one of the drugs targeting the FGFR1c/KLB complex that is currently in clinical trials. In the first-in-human clinical trial, a single subcutaneous injection of BFKB8488A to overweight or obese individuals was safe. BFKB8488A improved cardiometabolic parameters, such as a decrease in plasma triglycerides, LDL cholesterol, and fasting insulin, as well as reduced body weight and caloric intake (146).

Recently, the results of a phase 1b clinical trial using BFKB8488A (ClinicalTrials.gov Identifier: NCT03060538) in patients with T2D or NAFLD were published (131). The phase 1b clinical trial consisted of various dosing regimens (20-250 mg) and different dosing frequencies (weekly, every two weeks, and monthly) over 12 weeks to evaluate the safety and exploratory outcomes. The most common Grade 1 or 2 side effects of BFKB8488A treatment included gastrointestinal complications (43.8%), especially in the high-dose regimens (50%-100%), but no apparent loss of bone density (131). BFKB8488A treatment only resulted in slight weight loss in the high-exposure group (4.1%). While there were no apparent effects on insulin sensitivity markers, improvements were seen in plasma adiponectin, triglycerides, and high-density lipoprotein (HDL) cholesterol in BFKB8488A-treated patients in an exposure-dependent manner (131). Hepatic damage markers, among which plasma ALT, AST, and Pro-C3 levels, decreased in the medium- and high-exposure groups (131). Interestingly, BFKB8488A treatment decreased the liver fat fraction in patients with NAFLD by -13.0% from baseline in low exposure, -34.5% in medium exposure, and -49% in the high exposure group (131). The improvement in liver injury and steatosis is likely an indirect effect of BFKB8488A through its actions on adipose tissue, such as suppression of lipolysis. Overall, BFKB8488A treatment improved cardiometabolic parameters and markedly reduced liver fat, which makes this drug especially interesting for patients with NAFLD and NASH. Currently, BFKB8488A is in Phase 2 clinical trials for the treatment of NASH, which is expected to be completed in early 2023 (ClinicalTrials.gov Identifier: NTC04171765).

### 4.5 MK-3655 in clinical trials

Another FGFR1c/KLB-targeting compound currently in clinical trials, with no research published yet, is MK-3655 (NGM313, Merck Sharp & Dohme). In a phase 1 clinical trial (ClinicalTrials.gov Identifier: NCT03298465), one single administration of MK-3655 reduced fasting glucose levels and improved whole-body insulin sensitivity after 29 days in obese non-diabetic patients. After 36 days of treatment, liver fat content was reduced by >30% in 63% of patients receiving MK-3655, along with reductions in ALT, AST, plasma triglycerides, and LDL cholesterol (132). MK-3655 is currently tested for safety and efficacy in a phase 2 clinical trial in patients with pre-cirrhotic NASH (ClinicalTrials.gov Identifier: NTC04583423). Patients will receive a dose of MK-3655 once every four weeks, and effects on NASH resolution as one of the primary outcome measures will be evaluated after 52 weeks with liver fat content, fibrosis scoring, and NAS scoring as secondary outcome measures.

## 5 Conclusion

Preclinical studies have dramatically increased our understanding of KLB function over the last two decades. Nevertheless, the absence of *KLB* homozygous LoF carriers in extensive sequencing studies limits our ability to fully understand this gene’s function in humans. Given the extreme rarity of *KLB* homozygous LoF carriers, the *KLB* gene appears vital for embryonic development. This also implies that the genetic contribution of *KLB* to disease is most likely through heterozygous or oligogenic inheritance models. In line with this view, several heterozygous missense variants in the *KLB* gene are associated with various phenotypes and diseases. However, the effect of these missense variants on KLB protein function is somewhat conflicting and requires further functional analysis. In addition, the relevance of genetic *KLB* variants regarding the efficacy of KLB-targeting drugs remains to be established. Related to this, various pathophysiological factors appear to decrease KLB expression, which may also impact the therapeutic activity of FGF-based drugs. Developing FGF analogs with higher KLB binding affinity may bypass these issues. The feasibility of this approach has recently been demonstrated by showing that the introduction of mutations in the C-terminal tail of FGF21 (R203W/L194F) caused a tenfold increase in KLB binding affinity (63).

Although human bulk tissue mRNA *KLB* transcriptome datasets may give the impression that *KLB* is primarily expressed in adipose, liver, and pancreas, more in-depth analyses show that *KLB* transcripts are also present in many other tissues such as breast tissue, testis, lung, stomach, and intestine. These findings indicate that KLB might have other functions besides its role in metabolic regulation. However, little is known about the function of KLB in these tissues and whether FGF-based drugs can target them. The expression of *KLB* in the stomach and intestine may provide a simple explanation for the gastrointestinal side effects that occur after treatment with FGF-based drugs. While these gastrointestinal side effects have also been attributed to central FGF effects, evidence for *KLB* expression in the human brain is scarce. Aside from having potentially broader physiological functions, evidence is increasing that KLB may be multifunctional at a molecular level. Next to its role as an obligatory high-affinity coreceptor for FGF19/FGF21, recent evidence indicates that KLB can also impact FGFR4 receptor stability, tight junction complex formation, and ubiquitin-dependent lysosomal degradation of GP130. As these functions appear to be independent of its coreceptor function, it is conceivable that KLB operates in other subcellular compartments, such as the ER.

The recognition of KLB as an exciting drug target for a variety of common metabolic diseases encouraged many pharmaceutical companies to develop a multitude of FGF19 and FGF21 analogs. Aside from FGF19- and FGF21-based drugs, FGFR1c/KLB-targeting antibodies have also been developed as an alternative to the FGF21-mimetics. By directly targeting this receptor complex, global FGFR activation can be avoided. Although the pharmacokinetic and pharmacodynamic properties of FGF19 analogs, FGF21 analogs, and FGFR1c/KLB-targeting antibodies differ substantially, they all can target KLB. Many of these analogs have already been explored in late-stage clinical trials and have thus been tested on many patients. Despite their different pharmacokinetic and pharmacodynamic properties, most analogs show surprisingly similar effects on disease parameters, indicating an overlapping mode of action. Thus far, clinical trials have generated disappointing results concerning improving glycemic control, making it unlikely that FGF19/FGF21 mimetics will serve as a stand-alone treatment for T2D. Nonetheless, even short-term treatment with FGF19/FGF21 mimetics or FGFR1c/KLB-targeting antibodies results in remarkable reductions of liver fat content and markers of liver damage and fibrosis. Consequently, KLB-targeting drugs are particularly interesting as a therapeutic strategy for patients with NAFLD. However, as evidenced in this review, the successful application of these drugs will strongly depend on continued efforts to improve our understanding of human FGFR/KLB biology.

## Author contributions

AA, CV, JJ and DS contributed equally to this review. All authors contributed to the article and approved the submitted version.
